# The influence of anatomy app use on chiropractic students’ learning outcomes: a randomised controlled trial

**DOI:** 10.1186/s12998-016-0125-8

**Published:** 2016-12-01

**Authors:** Amanda J. Meyer, Norman J. Stomski, C. Dominique Losco, Anthony J. Armson

**Affiliations:** School of Health Professions, Murdoch University, 90 South St, Murdoch, WA 6150 Australia

**Keywords:** Chiropractic, Anatomy, Student, App, Learning outcomes, Randomised controlled trial

## Abstract

**Background:**

Anatomy apps supplement traditional learning; however, it is unknown if their use can improve students’ outcome. The present study examined whether the use of anatomy apps improved student performance on a neuroanatomy assessment.

**Methods:**

Second-year anatomy students, enrolled in a Bachelor of Science with Chiropractic Major program, were randomly allocated to experimental and control groups in July 2015. Students completed the Self-Directed Learning Readiness Scale (SDLRS). The experimental group had access to iPads with four anatomy apps for three weekly classes (1.5 h each). One week after the last class, students were assessed by an online 30-question neuroanatomy test. Linear regression was used to examine the association between test scores and app use, gender, previous anatomy unit score and SDLRS scores. Students’ views on apps were collected by focus group discussion immediately after the test.

**Results:**

Completed questionnaires were obtained from *n* = 25 control and *n* = 25 experimental students. There was no association between app use and neuroanatomy assessment score (B = 1.75, 95 % CI: -0.340-3.840, *p* = 0.099). Only previous anatomy unit score (B = 0.348, 95 % CI: 0.214-0.483, *p* < 0.001) affected neuroanatomy assessment scores. Students favored apps with clinical images and features including identification pins, sliding bars and rotatable 3D images.

**Conclusions:**

App use did not enhance learning outcomes in a second-year anatomy unit.

## Background

Teaching in anatomy is evolving, with a reduction in contact hours, increased student numbers, increased costs associated with cadavers and advances in technology, all driving change in the anatomy learning environment [1, 2]. The use of advancing technology in anatomy education is supported, but it is noted that these tools are to complement how students explore, learn and collaborate in their learning environments and not to replace the existing practices [3–5]. A previous study examining the effect of a 3D neuroanatomical teaching tool (MRI data sets with 3D neuroanatomical structures overlaid) reported that 79 % of experimental students strongly agreed that it helped to visualize 3D structures and spatial relationships in the brain [6]. The majority (64 %) of students in that study reported they would have preferred user control [6]. Therefore, self-directed use of anatomy apps may improve students’ learning outcomes on a neuroanatomy assessment.

Computerized 3D teaching tools in anatomy education are not new, but they are now contained in mobile software applications (apps) that are easily accessible to students. Mobile technology devices (smartphones and tablets) are owned by the majority of students enrolled in anatomy units at Murdoch University [7]. Two-thirds of students owned one or more anatomy apps however the majority used apps for less than 30 min per week, which suggests that it may be beneficial to introduce strategies that enhance the use of anatomy apps [7]. Hence, this study was designed to encourage a group of students to use anatomy apps in a loosely-guided self-directed manner during class time and examine whether it improved student outcomes on a summative neuroanatomy assessment.

## Methods

### Sample

Fifty seven students in a Bachelor of Science with Chiropractic Major program (chiropractic major: *n* = 53; biomedical major: *n* = 2; exercise physiology major: *n* = 2) enrolled in CHI282 Human Anatomy II at Murdoch University (Semester 2, 2015) were randomly allocated to experimental (*n* = 31) and control (*n* = 26) groups in July 2015. A random number generator was used to create a randomization list. The group allocation was placed in sequentially numbered, opaque, sealed, envelopes. Research staff not involved with teaching the students handed the envelopes to students, who then immediately opened the envelope and notified the staff of group allocation.

All students were asked to complete the Self-directed Learning Readiness Scale (SDLRS) questionnaire during their first gross anatomy laboratory session. The SDLRS is a validated self-report instrument to assess students’ readiness for self-directed learning [8]. Originally designed to measure the readiness of undergraduate nursing students for self-directed learning [8], the scale has since been used in a number of undergraduate educational settings such as medicine [9, 10]; paramedicine [11] and pharmacy [12]. The SDLRS questionnaire is a reliable and valid scale with 40-items rated on a five-point Likert scale from ‘strongly disagree’ to ‘strongly agree [8, 13]. The maximum score is 200 and a score greater than 150 indicates that students have a high readiness for self-directed learning [8, 13].

### Intervention

Students in the experimental group were given access to eight iPad© mini 2 tablets pre-loaded with neuroanatomy apps for three loosely-structured (learning objectives provided in the unit syllabus) gross anatomy laboratory sessions (1.5 h each). Each device had the following neuroanatomy apps: Brain and Nervous System Pro III [14], Essential Anatomy 5 [15]; Brain and Nervous Anatomy Atlas: Essential Reference for Students and Healthcare Professionals [16] and iSurf BrainView [17].

During the first session, experimental students were given access to the apps and were suggested to identify 55 structures in matching coronal sections of a cadaver brain and a T1-weighted MRI. During the second session, some students used the available apps to help construct a plasticine brainstem model. The third session was mainly focused on the pathways of the spinal cord tracts, for which no app was particularly suitable.

Students in the control group attended the three gross anatomy laboratory sessions and had access to all the same resources except the iPads.

### Anatomy assessment

Students’ knowledge of neuroanatomy was assessed using a closed-book 30 question image-based multiple choice test using the Moodle (Moodle Pty Ltd, Perth, Western Australia) quiz function. Students had 30 min to complete the summative assessment which was administered seven days after the third laboratory session. As this unit is the third and last anatomy unit taken by students, the majority of questions were higher-order (Levels 3 and 4) of the Blooming Anatomy Tool [18]. The assessment was marked automatically by Moodle Quiz. Marks were downloaded, and then matched with the SLDRS score and group number and then de-identified data prior to analysis.

### Focus group

A focus group was conducted immediately after the summative assessment by a staff member who was not involved in the teaching of anatomy to the students. The focus group was held for 60 mins with seven students and was audio-recorded and notes were taken for clarification. To stimulate discussion, students were asked the following questions: (1) Do you think the use of apps enhanced your learning outcomes in the neuroanatomy wet labs? (2) Do you think there were any limitations of the apps that affected your learning outcomes in the neuroanatomy wet labs? (3) Which particular apps did you find most useful? Why? (4) Which particular apps did you find least useful? Why? and (5) Did you download and use any apps outside of the anatomy laboratory?

### Statistical analysis

Data were analyzed using IBM® SPSS® Statistics package, version 21. All data were reported descriptively. Linear regression was used to examine the association between anatomy app use and neuroanatomy assessment scores. The students’ gender, previous anatomy unit scores, and SDLRS scores were entered into the regression model as potential confounder factors as they had been found to influence educational app use in previous studies [19–21]. Cronbach’s alpha was derived for the SDLRS scale.

## Results

### Participants

Completed questionnaires were obtained from 50 out of 57 student enrolled in the study. Two students did not consent to have their data included and five students had incomplete data. Participant characteristics are reported below in Tables 1 and 2.

**Table 1:** Neuroanatomy apps used in the gross anatomy laboratory

Name of app	Developer	Version/Size	Positives	Negatives
Brain and Nervous Anatomy Atlas: Essential Reference for Students and Healthcare Professionals	Visible Body	6.0.11/458 MB	3D Rotation One axial section Identify spinal cord tracts	Cost ($9.99) No spinal cord pathways
Brain and Nervous System Pro	3D4Medical.com, LLC	3.8/758 MB	Pins Ability to slice through brain Axial/sagittal/coronal views Sliding bar to easily advance through multiple images	Cost ($9.99) No spinal cord pathways
Essential Anatomy 5	3D4Medical.com, LLC	5.0/645 MB	3D Rotation Isolate/Hide structures Information attached to structure	Cost ($24.99) Needed to hide all of the cranium to get to brain Quiz function very basic No spinal cord pathways
iSurf BrainView	Netfilter	4.1.0/30.4 MB	Free MRI Axial/sagittal/coronal views Sliding bar	Not all structures are labeled

**Table 2:** Characteristics of the study participants

	Experimental (*n* = 25)	Control (*n* = 25)	*p* value
Mean age (years)	22, SD 4	22, SD 6	0.954
Males (%)	12 (50 %)	10 (40 %)	0.569
Mean previous anatomy unit score (%)	66, SD 8 %	65, SD 9 %	0.557

*SD* standard deviation

### Quantitative outcomes

#### Self-directed learning readiness scale

Cronbach’s alpha for the SDLRS total scale score was 0.88. The mean total SDLRS score, administered prior to the intervention, was not different between the groups (control: 150.1, SD 13.5; experimental: 151.4, SD 12.0; *p* = 0.716).

#### Intervention outcome

The mean neuroanatomy assessments scores were not different between the groups (control: 63.3 %, SD 16.7 %; experimental: 70.0 %, SD 16.7 %; *p* =0.106). Table 3 displays the results of the linear regression. Neither self-directed laboratory-based anatomy app use, gender, nor SDLRS affected neuroanatomy assessment scores. The only factor that influenced neuroanatomy assessment scores was previous gross anatomy unit score (B = 0.348, 95 % CI: 0.214-0.483, *p* < 0.001). The overall model fit was R^2^ = 0.472.

**Table 3:** Results of the multivariate linear regression analysis

Variable	Unstandardised B coefficient	95 % CI for unstandardised B coefficient	*p* value
Previous anatomy unit score	0.348	0.214 – 0.483	<0.001
Group Allocation	1.75	−0.340 – 3.84	0.099
Gender	−0.754	−2.97 – 1.46	0.496
SDLRS score	−0.005	−0.099 – 0.090	0.920

### Qualitative outcomes

#### Focus group data

Four common themes emerged from the focus group discussion:

Students’ comments on the features of individual apps: Students highly praised the inclusion of identification pins, sliding bars to advance through sections, isolate/hide functions, ability to rotate 3D structures and quizzes for revision in anatomy apps. In particular, students said apps were useful for identifying and understanding the form of c-shaped structures in the brain. Lastly, students noted that app quiz functions were very basic and not at the same knowledge level as their summative assessment.

Students’ comments on the use of individual apps: Students reported that they would have like more guidance on how to use the apps (training/familiarisation prior to session), and even reported that they would have preferred a tutor-led session (tutor instruction to the group using the apps) rather than being self-directed. Students admitted that they did not use anatomy apps at home as they were not prepared to pay for them on their own devices. Some students stated that they did not use anatomy apps much in class as it took time away from viewing cadavers which they knew would be on the summative assessment. Students acknowledged that they preferred using apps on tablets/computers rather than on smartphones due to a larger screen size and increased image resolution.

Students’ comments on the content and graphics in apps: Students reported that the apps were helpful for identifying structures in specimens and clinical images that were not labelled in the anatomy laboratory. The graphics, particularly in the 3D4medical.com apps were of high quality and were easily manipulated through the touch screens. All the anatomy apps investigated lacked sufficient detail for spinal cord pathways and lesions, which were important concepts covered in the summative assessment.

Students’ comments on the price of apps: Students noted that the price of apps was prohibitive (“…too expensive to purchase!”), however students also reported that they did not download free apps even when suggested by the course instructor.

## Discussion

Self-directed use of neuroanatomy apps during a limited number of anatomy laboratory sessions was not significantly associated with students’ performance on a neuroanatomy assessment. Students may have encountered a “technology learning curve” [19], whereby time learning to use the apps may have taken time away from learning the neuroanatomy. Additionally, the apps are limited to Level 1 (knowledge) and level 2 (comprehension) of the Blooming Anatomy Tool [18], whereas the summative neuroanatomy assessment contained many questions from Level 3 (application) and Level 4 (analysis).

In contrast to the present study, a recent study reported that anatomy students perceived that an iPad educational intervention improved laboratory and lecture performance [22]. However, this perceived improvement was based on the students’ subjective views about their participation in lectures and laboratory sessions, and no pre/post intervention summative assessments were undertaken. Whether the intervention used in that study influences objective assessments needs to be established in further studies [22].

Another recent study found that the use of a digital interactive book, provided on an iPad, significantly improved anatomy students’ summative assessment scores post resource [23]. However, that study differed from the present study since it was non-randomized, did not use a control group, and did not control for the influence of either grade point average or demographic factors [23]. Hence, these differences in research methods may account for the inconsistent findings between studies.

The only factor that influenced neuroanatomy assessment scores was students’ previous gross anatomy unit score. Earlier reports also demonstrated that previous academic performance was shown to have a statistically significant relationship with student performance [24, 25]. Therefore, academics may be able to identify students at risk of failing due to past performance.

There was no association between SDLRS and neuroanatomy assessment scores in the present study. A weak correlation between SDLRS scores and academic performance has previously been reported in a study of 130 first year medical students in India [10]. Similarly, SDLRS scores were not related to knowledge levels in a study of 124 health profession students in the United States [26]. SDLRS score may not be an important predictor of academic performance when students are given learning objectives and open access to learning resources.

A number of limitations of the current study should be noted. Firstly, the neuroanatomy module comprised the first three weeks of the teaching semester which reduced the number of laboratory sessions where students had access to apps. Secondly students mostly used the apps during the initial laboratory session, and used them less during subsequent sessions as they did not have time to get over the “technology learning curve” before the summative assessment. Hence, it would be worthwhile to include “time spent using apps” as independent variable in further studies. Lastly, this study drew participants enrolled in one unit at a single institution who were mostly from one major which may limit the generalizability of our findings. A longer, larger, cross-institutional study may help to clarify whether use of anatomy apps can influence students’ learning outcomes.

The practical implications for integrating the use of anatomy apps in the laboratory are five-fold: (1) anatomy instructors should be able to demonstrate how to use apps in a seamless, readily comprehensible, manner; (2) an adequate amount of time should be provided to students to familiarize themselves with the apps prior to use in the laboratory; (3) students can readily utilize apps for revision of basic anatomy; (4) 3D apps are particularly useful for identifying and understanding the form of c-shaped structures, such as the caudate nucleus and fornix and (5) there is a need for higher-order knowledge quizzes and inclusion of spinal cord pathways in apps featuring neuroanatomy.

## Conclusion

Our findings indicated that short-term use of anatomy apps did not influence student learning outcomes. Given the limited number of studies conducted in this area, further studies are warranted to clarify whether use of anatomy apps can influence students’ learning outcomes. In particular, such studies should examine whether time spent using educational apps, extent of instruction in using educational apps, and matching app content to learning outcomes, significantly influences anatomy learning outcomes.

## Abbreviation

SDLRS: Self-directed learning readiness scale
